# Targeting Tumor-Associated Macrophages in Anti-Cancer Therapies: Convincing the Traitors to Do the Right Thing

**DOI:** 10.3390/jcm9103226

**Published:** 2020-10-08

**Authors:** Cristina Belgiovine, Elisabeth Digifico, Clément Anfray, Aldo Ummarino, Fernando Torres Andón

**Affiliations:** 1 Humanitas Clinical and Research Center—IRCCS, Via Manzoni 56, 20089 Rozzano, Milan, Italy; elisabeth.digifico@humanitasresearch.it (E.D.); clement.anfray@humanitasresearch.it (C.A.); 2 Department of Biomedical Science, Humanitas University, Via Rita Levi Montalcini 4, 20090 Pieve Emanuele, Milan, Italy; aldo.ummarino@hunimed.eu; 3 Center for Research in Molecular Medicine & Chronic Diseases (CiMUS), Universidade de Santiago de Compostela, 15706 Campus Vida, Santiago de Compostela, Spain

**Keywords:** TAM, reprogramming of TAM, anti-cancer treatment, immune landscape, immunotherapy.

## Abstract

In the last decade, it has been well-established that tumor-infiltrating myeloid cells fuel not only the process of carcinogenesis through cancer-related inflammation mechanisms, but also tumor progression, invasion, and metastasis. In particular, tumor-associated macrophages (TAMs) are the most abundant leucocyte subset in many cancers and play a major role in the creation of a protective niche for tumor cells. Their ability to generate an immune-suppressive environment is crucial to escape the immune system and to allow the tumor to proliferate and metastasize to distant sites. Conventional therapies, including chemotherapy and radiotherapy, are often not able to limit cancer growth due to the presence of pro-tumoral TAMs; these are also responsible for the failure of novel immunotherapies based on immune-checkpoint inhibition. Several novel therapeutic strategies have been implemented to deplete TAMs; however, more recent approaches aim to use TAMs themselves as weapons to fight cancer. Exploiting their functional plasticity, the reprogramming of TAMs aims to convert immunosuppressive and pro-tumoral macrophages into immunostimulatory and anti-tumor cytotoxic effector cells. This shift eventually leads to the reconstitution of a reactive immune landscape able to destroy the tumor. In this review, we summarize the current knowledge on strategies able to reprogram TAMs with single as well as combination therapies.

## 1. Introduction

Macrophages are specialized phagocytic cells of the innate immune system. They belong to the mononuclear phagocyte system, comprising both tissue resident macrophages and circulating monocytes, which are available to be recruited at sites of inflammation and tissue damage, such as tumors. Plasticity is one of the main features of macrophages, since they display a broad spectrum of activation states with distinctive phenotypes and functions. Differentiating monocytes, reaching the tissues, can meet and adapt to particular local stimuli able to activate distinct genetic programs [1,2,3,4,5].

In this broad spectrum of activation states, two polarized extremes have been defined: the M1 (or classically activated, pro-inflammatory/anti-tumoral) macrophages and the M2 (or alternatively activated, anti-inflammatory/pro-tumoral). Prototypical M1 macrophages are activated by lipopolysaccharides (LPS) and the pro-inflammatory cytokine IFN-γ. M1-like macrophages are able to neutralize bacterial infections and produce pro-inflammatory cytokines (e.g., IL-1β, TNF-α, and IL-12). They are able to kill cancer cells, inhibit angiogenesis, and promote adaptive immune responses. As opposite, prototypical M2 macrophages are induced by the anti-inflammatory cytokines IL-4 and IL-13. They can suppress Th1 immunity, are central effectors in the healing of injured tissues, and promote tumor progression and neo-angiogenesis. The uncontrolled and prolonged activation of inflammatory macrophages could represent a risk for the body, therefore these cells typically shift towards an M2 phenotype over time [3,5]. Although it has been recognized that a complex spectrum of activation states exists for macrophages in cancer, depending on the type of tumor and their particular localization (i.e., periphery versus centre of the tumor), especially at advanced stages, these cells most commonly acquire an M2-like phenotype.

Tumor-associated macrophages (TAMs), presenting an M2-like polarization, inhibit immuno-stimulatory signals and lack cytotoxic activity, therefore promoting tumor development and survival [3]. TAMs are macrophages, which have been shaped by tumor-derived factors to promote cancer progression. These corrupted cells are responsible for progression and resistant to conventional antitumor treatments, such as chemotherapy or radiotherapy, but also to the latest immunotherapies, such as anti-PD1 [3,6,7,8].

For these reasons, TAMs are promising targets for novel anti-tumor treatments. Several therapeutic approaches have been assayed to deplete TAMs in tumors; however, new approaches are majorly focused on the exploitation of TAMs themselves as weapons to fight cancer. The reprogramming of TAMs aims to convert immunesuppressive and pro-tumoral macrophages (M2-like) into immunostimulatory and anti-tumor cytotoxic effector cells (M1-like). If effective and long-lasting, this switch is expected to reconstitute a reactive immune system with the ability to fight and completely eliminate the cancer in the patient. In this review, we summarize the current knowledge on the role of macrophages in tumors and strategies to re-educate TAMs. 

## 2. Role of Macrophages in Tumors

**Figure 1:**
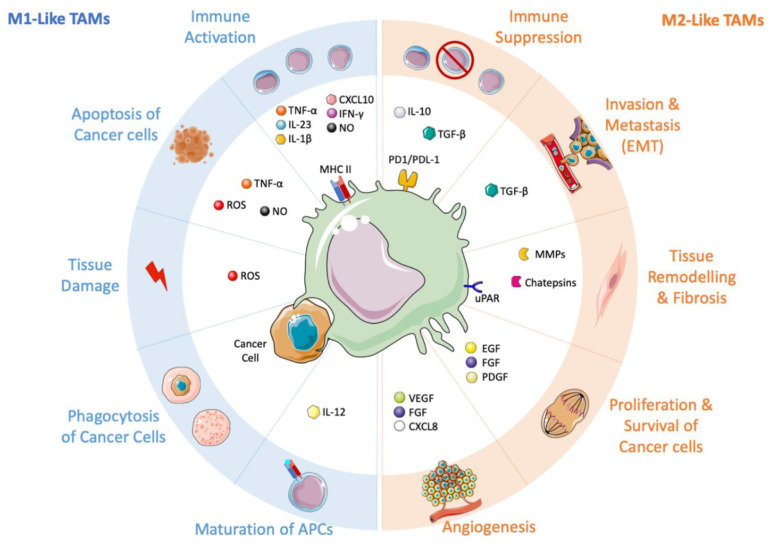
Tumor-associated macrophages (TAMs) and their ambivalent role in shaping the tumor microenvironment. On the left side, the anti-tumoral M1-like macrophages, stimulated by immunostimulatory cytokines (e.g., IL-1β, IL-12, IL-23, TNF-α, IFN-γ). M1-like TAMs promote the recruitment and activation of T cells by producing CXCL10, TNF-α, and other cytokines. Through the release of TNF-α, ROS (Reactive Oxign Species), and NO, they can directly kill tumor cells. M1-like macrophages induce tissue damage, maturation of APCs (Antigen Presenting Cell) and they can actively phagocytose cancer cells. On the right side, the pro-tumoral M2-like macrophages, release immuno-suppressive mediators, such as IL-10, TGF-β, IDO1/2, which support regulatory T cells. These pro-tumoral immune cells promote tumor proliferation (EGF, FGF, PDGF), angiogenesis (CXCL8, VEGF, FGF), invasion and metastasis (TGF-β), and a continuous tissue remodeling (MMPs, cathepsins, uPAR).

Tumor-associated macrophages can represent up to 50% of the tumor mass, being the main immune population in solid tumors. They can derive from circulating monocytes and tissue resident macrophages. Specific signaling molecules, such as CCL2, CSF-1, cytokines, and complement components (i.e., C5), are able to rapidly recruit circulating inflammatory monocytes at sites of tumor growth [3]. However, TAMs can also derive directly from resident macrophages, originally present in the healthy tissue later developing cancer. The tumor microenvironment can shape TAMs’ behavior through the release of different stimuli, which typically shift the macrophages towards an immunosuppressive pro-tumoral phenotype, or, rarely, towards a pro-inflammatory and anti-tumoral phenotype (Figure 1) [3,9,10]. Thus, macrophages can play a dual role in the development of different tumor types [11], and their number and polarization status has been associated with a better or worse patient survival. In several tumor types, such as osteosarcoma and esophageal tumors, their presence is associated with better overall survival and longer metastasis progression-free survival [12,13]; instead, in other tumors, macrophages are associated with worse prognosis, especially when linked to low numbers of CD8+ cells, the lymphoid cellular type responsible for the killing of tumor cells [14,15,16,17].

TAMs showing M2-like features are typically associated and responsible for the bad prognosis of the disease, and for this reason, they could be considered the corrupted policemen of our immune system [3,18]. They are implicated in the initiation and progression of the tumor, through the secretion of signaling molecules, such as transforming growth factor beta (TGF-β), vascular endothelial growth factor (VEGF), macrophage colony-stimulating factor (M-CSF), interleukins or chemokines (IL-10, IL-6, and CXCL-8) [19,20,21], and extracellular vesicles (EV) with immunosuppressive properties [22].

TAMs promote tumor invasion and metastasis through the secretion of matrix metalloproteases, serine proteases, and cathepsins. Due to the release of these factors, the cell–cell junctions and the basal membrane are disrupted [23]. Several molecules are involved in the remodeling of the extracellular matrix. IL-4 induces the protease activity of cathepsins that promotes breast cancer invasion and metastasis [24]; other factors secreted by TAMs, such as TGF-β, VEGF, CCL8, COX-2, SPARC, MMP9, and MMP2 contribute to the metastatic properties of cancer cells [25,26,27,28,29,30]. TAMs play a pivotal role also in the process of epithelial to mesenchymal transition (EMT). This process promotes tumor invasion and metastasis through the reduction of epithelial markers, such as E-cadherin, and the induction of mesenchymal markers, such as vimentin, slug, snail, and fibronectin [31]. The TLR4/IL-10 pathway, TGF-β, and CCL18 produced by TAMs are associated with EMT [32,33,34,35,36]. Moreover, TAMs are implicated in the sustainment of cancer stem cells (CSC). CSCs are a population of tumor cells, which share some features with stem cells, being able to initiate tumorigenesis thanks to their ability for continuous self-renewal and differentiation [37]. In this context, TAMs produce soluble factors (e.g., TGF-β, IL-6) that promote survival of CSCs [20,21,38,39]. Our group has recently found that also GPNMB produced by macrophages induces cancer stemness via CD44 binding and release of IL-33 [40].

Another pro-tumoral function of TAMs is related to their ability to induce and sustain angiogenesis, supporting the formation of tumor vessels. Angiogenesis is necessary to sustain tumor growth and progression because neo-vessels bring oxygen and nutrients to the tumor. TAMs produce several factors that contribute to create new vessels: VEGF, TGF-β, CXCL8, PDGF but also MMP9 and TIE2 (endothelial-specific receptor tyrosine kinase) [19,41,42,43,44,45]. New vascularization is activated by the binding of TIE2 with Angiopoietin 2 (ANG2); in hypoxic conditions, both the ligand (ANG2) and the receptor (TIE2) are overexpressed [46,47].

The presence of TAMs is frequently associated with the failure of antitumoral treatments, such as chemo- and radiotherapy [48,49]. The resistance to these therapies can be mediated by the release of cytokines from TAMs; in particular, IL-6 has been demonstrated to induce tolerance in the treatment of breast [50], pancreatic [51], and colorectal cancer [52,53]. TAMs-derived cathepsins B and S were able to prevent paclitaxel-induced tumor cell death in mammary carcinoma [54]. In prostate cancer, IL-4 impaired the efficacy of radiotherapy by inducing the CSFR1 signaling pathway [55]. Accordingly, depletion of macrophages, through different approaches, has been demonstrated to increase the efficacy of several conventional drugs [48].

TAMs can impair antitumoral immunotherapy thanks to their ability to shape the immune system and polarize the tumor environment towards an immunosuppressive status. In fact, TAMs are able to inhibit CD4+ and CD8+ effector function via the release of cytokines, chemokines, and enzymes (e.g., CCL2, IL-10, TGF-β, Cathepsin K, COX-2, and MMPs); besides, TAMs activate the T-reg subpopulation, impairing the effector function of lymphocytes. Macrophages in tumors express also inhibitory checkpoint receptors (PD-L1/2, PD-1, CD80, CD86, and VISTA) frequently associated with immunotherapy failure [56]. For example, TAMs overexpress PD-L1 that reduces the efficacy of anti PD-1 therapies [57], by sequestering anti-PD-1 antibodies and also by unspecific binding [58].

## 3. Presence of TAMs in Different Tumor Types

Taking into account the properties of macrophages described in the previous paragraph, it is now commonly accepted that high numbers of TAMs with an M2-like anti-inflammatory phenotype are typically associated with poor patient outcome, while TAMs polarization towards an M1-like pro-inflammatory phenotype tend to correlate with favorable prognosis and longer survival (Table 1) [59,60,61,62,63]. Below, we address some of the features related to macrophage polarization in different tumor types.

## 4. Malignant Pleural Mesothelioma

Malignant Pleural Mesothelioma (MPM) is a very aggressive type of cancer, characterized by a chronic inflammation, commonly caused by inhalation of asbestos fibers. Macrophages are key drivers of this chronic non-resolving inflammation in the attempt to clear away the non-degradable asbestos fibers [64,65]. Since the early steps of tumorigenesis, cancer and stromal cells increase the number of TAMs in the tumor, by producing chemokines and growth factors (e.g., CCL2 and CSF1) [3,66,67]. Furthermore, in mesothelioma, the tumor cells influence also their differentiation into immune-suppressive and tumor-promoting macrophages [3]. It has been demonstrated that a high density of CD68+ macrophages in surgical mesothelioma samples was correlated with worse patient clinical outcome [68,69,70] and their depletion with zoledronic acid strongly reduced tumor growth in mouse mesothelioma models [71]. Thus, macrophages in the tumor microenvironment of human mesothelioma are correlated with faster and more aggressive tumor growth. Moreover, Cornellisen et al. demonstrated that the CD163+ macrophages/total TAM ratio could be used as a prognostic marker of local tumor outgrowth (LTO), a common complication in MPM after invasive procedure. They found also that patients with this outgrowth show a significantly lower number of CD8+ cells, compared with patients who did not develop LTO [72].

## 5. Gliomas

In gliomas, the TAMs population is constituted by both microglia and newly recruited monocyte-derived macrophages able to influence tumor development [87]. They can represent up to 40% of the cells in the tumor mass, underlying their importance in shaping the immunosuppressive microenvironment of these aggressive tumors [87,88,89]. Interestingly, grade IV gliomas have a higher density of TAMs compared with grade II and III. Komohara et al. [73] demonstrated not only that the number of macrophages correlates with the grade of malignancy, but also that activation of macrophages towards the M2-like phenotype is correlated with higher histological grade. Moreover, Sorensen et al. demonstrated that M2-like TAMs are associated with more aggressive tumors and can predict worse prognosis in high-grade glioma [74]. High gene expression ratio of CD163/CCL3 in gliomas, as M2 and M1 macrophage markers, respectively, and PD-1+ CD4 T cells in the blood of tumor patients were associated with poor prognosis [75]. Increased numbers of CD68+ and higher ratio of CD163/AIF+ cells, as TAMs markers, and more FOXP3+ cells were associated with shorter progression-free survival, while high CD3+ and CD8+ T cells accompanied by low CD68+ and high IDO+ cell counts were associated with better glioma prognosis [76].

## 6. Lung Cancer

Several clinical studies focused on TAMs have revealed the controversial role of macrophages in lung cancer. The use of only generic markers for macrophages, such as CD68, has led to mixed results on the prognostic value of macrophages in these types of tumors. Furthermore, macrophages can be spread in different tissue compartments in the lung, such as tumor stroma, tumor islets, and alveolar space; indeed, the macrophages distributed in multiple tissue locations may display different biological properties [11,59,60,61,62,90]. Several studies showed that high levels of CD68+ macrophages in tumor cell islets were associated with a longer survival in non-small-cell lung cancer (NSCLC) [61,91,92,93,94]. Opposite to this, high level of macrophages in the lung tumor stroma were correlated with reduced survival [61,91,92]. Jackute et al. performed a double immunohistochemical staining on lung tissue samples from NSCLC patients: CD68/iNOS, for M1 macrophages, and CD68/CD163, for M2 macrophages. Their analysis revealed that a high level of M1 macrophages in the tumor islets together with a low level of total tumor-infiltrating M2 macrophages were correlated with improved patients’ survival [77]. Moreover, Sumitomo et al. demonstrated that stromal TAMs density in the lungs is associated with lymph node metastasis and reduced overall survival [95], as well as the density of alveolar macrophages with M2 phenotype (CD163+) was associated with reduced disease free and overall survival. High density of M1 macrophages, characterized by CXCL9, CXCL10, and STAT1 activation, accompanied by the presence of resident memory T cells was also strongly associated with better outcomes in patients with lung cancer [96].

## 7. Pancreatic Cancer

Acute pancreatitis are predominantly infiltrated by M1-like, pro-inflammatory macrophages, while chronic pancreatitis are mostly infiltrated by M2-like, anti-inflammatory macrophages. Interestingly, it has been observed that M2-like macrophages CD68/CD204+ are more abundant in patients with pancreatic cancer compared with patients with chronic pancreatitis, and their number was correlated with larger tumor size and shorter survival in patients with pancreatic cancer [78,97,98]. Thus, providing a clear indication for the harmful role of TAMs in this type of cancer.

## 8. Colorectal Cancer

In contrast with other solid tumors, numerous studies have demonstrated that TAMs may present a protective role in colorectal cancer [82,99,100,101]. High macrophage infiltration at the tumor front was correlated with improved survival in colorectal cancer patients, in part due to their antitumor action [82,99]. Furthermore, TAMs infiltration at the invasive front was also associated with reduced hepatic metastasis [83]. Despite this evidence, other studies demonstrated the pro-tumoral role of TAMs in colorectal cancer. Liu et al. showed that a high Wnt5a+CD68+/CD68+ TAMs ratio can be associated with poor prognosis in colorectal cancer patients. Moreover, they revealed that Wnt5a could induce an M2-like polarization of TAMs, promoting tumor growth and metastasis [84]. It was also demonstrated that TAMs in colorectal cancer are able to promote angiogenesis and metastasis through the secretion of VEGF [102]. Bailey et al. showed that macrophage count in the total tumor area was a bad prognosis indicator, and macrophage numbers significantly increase with tumor stage [103]. As a whole, these studies highlight the controversial role of macrophages in colorectal cancer, which may be explained by the different localization of macrophages within the tumor tissue. Indeed, macrophages at the invasive front are commonly anti-tumoral, since they are less exposed to tumor-derived cytokines and are located in less hypoxic areas; thus, averting their pro-tumoral/anti-inflammatory (M2-like) differentiation [104]. Moreover, also nutrients and microbiota are able to shape the function of intestinal macrophages, key players in the maintenance of gut homeostasis. Wrong dietary habits, together with an alteration of microbiota composition, can cause intestinal chronic inflammation and, finally, colorectal cancer. Furthermore, a disruption of the IL-10/IL-10R axis, a key player in the regulation of intestinal macrophages, can increase macrophage expression of pro-inflammatory mediators, leading to intestinal inflammation [105].

## 9. Breast Cancer

Despite the great heterogeneity of breast cancer subtypes, the presence of TAMs usually correlates with poor prognosis [79,80,106]. High-grade hormone receptor negative tumors (basal-like subtypes), which present a very poor clinical outcome, have been associated with a tumor microenvironment rich in TAMs [81,107], and their role in tumor progression is often linked with the induction of EMT [36,107]. Su et al. demonstrated that mesenchymal-like breast cancer cells are able to polarize macrophages towards TAMs by producing GM-CSF. This shift was associated with the increase in CCL18+ macrophages [36], higher number of metastasis, and reduced patient survival [26]. Furthermore, it has been found also in triple-negative breast cancer that the overexpression of EMT-related AXL kinase is able to promote TAMs polarization, and it is correlated with poor prognosis [107]. A high density of M1 macrophages expressing iNOS in the center of the tumor, together with high presence of CD8+ cells, has been associated with improved survival in HER2+ breast cancer; in contrast, the presence of CD163+ macrophages and T-reg cells was linked to poor prognosis [17]. Fortis et al. have also demonstrated that high ratio between CD8+ and CD163+ cells evaluated in the tumor center, and the reverse low CD8+/CD163+ ratio in the tumor invasive margin, represent a valuable prognostic marker in breast cancer [16].

As a whole, these studies demonstrate that TAMs are essential for the survival and progression of most types of solid tumors [85,86,108], and their therapeutic targeting and reprogramming towards an antitumor M1-like phenotype is a promising approach to fight cancer [48,49,109].

## 10. Therapeutic Approaches for Macrophage Reprogramming

**Figure 2:**
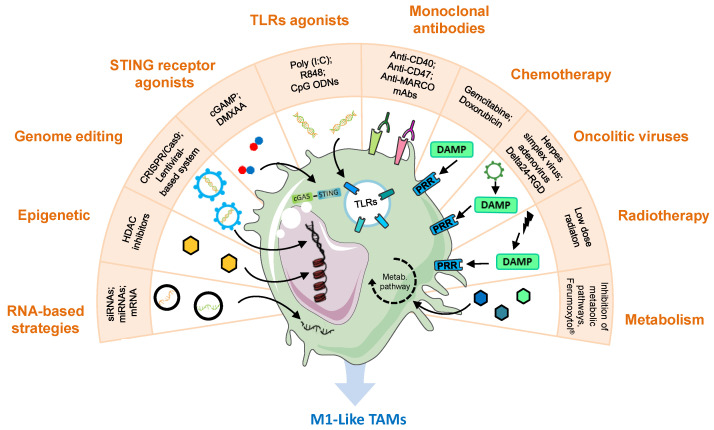
Reprogramming of tumor-associated macrophages is a promising target for novel anti-tumor treatments. This figure summarizes and gives examples of various strategies with this purpose. The inhibition of immunosuppressive genes has been investigated through the delivery of the CRISPR/Cas9 machinery or lentiviral vectors to directly edit TAMs genome, HDAC inhibitors for epigenetic regulation or nanoparticles encapsulating siRNAs, miRNAs, or mRNAs to manipulate gene transcription. TLR and STING agonists have been shown to reprogram TAMs towards an M1-like phenotype. Monoclonal antibodies targeting the CD47/SIRPα axis, activating CD40, or blocking the scavenger receptor MARCO activate the antitumoral functions of macrophages. Traditional chemotherapeutics such as gemcitabine or doxorubicin, oncolytic viruses, and low doses of radiation, which induce the release of DAMPs by tumor cells, have been reported to polarize macrophages towards M1-like phenotype, through pattern recognition receptor stimulation. Manipulation of TAMs metabolism has been modestly explored, showing good results in experimental settings through the inhibition of glycolysis, hypoxia, lactate, cholesterol, IDO, arginase, glutamine, or adenosine pathways in macrophages, but also by the administration of iron-based nanoparticles (NPs) (Ferumoxytol^®^) or oxygen. cGAMP, cyclic guanosine monophosphate–adenosine monophosphate; CpG ODNs, CpG oligodeoxynucleotides; DAMP, damage-associated molecular pattern; DMXAA, 5,6-dimethylxanthenone-4-acetic acid; HDAC, histone deacetylase; MARCO, macrophage receptor with collagenous structure; PRR, pattern recognition receptors; R848, resiquimod; STING, stimulator of interferon genes; TAM, tumor-associated macrophage; TLRs, toll-like receptors.

In cancer, TAMs therapeutic reprogramming is intended to switch their M2-like protumor properties towards M1-like macrophages, with active defensive activity and antitumor functions, including direct killing of tumor cells, inhibition of angiogenesis, normalization of tumor vessels, and improvement of adaptive immune responses [49]. Furthermore, TAMs reprogramming in the tumor microenvironment (TME) is also expected to synergize and boost the activity of other antitumoral treatments currently applied in the clinic, such as immune-checkpoint inhibitors (ICIs) or CAR T cells [8]. In the next sections, we show the whole scenario on TAM reprogramming, starting from the initial evidence of some cytotoxic drugs and low-dose radiotherapy towards a broad variety of pharmacological approaches and new drug delivery approaches (Figure 2).

## 11. Chemotherapy, Radiotherapy, and Oncolytic Virus Inducing Cancer Cell Death and TAMs Reprogramming

As a result of immunogenic cell death (ICD) induced by doxorubicin, macrophages become activated and contribute to the antitumoral effect of this cytotoxic drug [78,110]. Other chemotherapeutics of the anthracycline family, oxaliplatin and bortezomib, as well as radiation or photodynamic therapy, have also demonstrated induction of ICD and activation of the immune system to fight against the tumor [111]. Cancer cells killed by ICD expose calreticulin and other endoplasmic reticulum proteins; they release cytokines and damage-associated molecular patterns (DAMPs), such as ATP or HMGB1, and also tumor antigens, which stimulate antitumor immune responses, resulting in the recruitment and activation of macrophages and T cells to fight against the cancer cells. For other chemotherapeutic drugs, such as gemcitabine, the results are not so clear, and their impact on TAMs’ polarization may depend on the tumor type or on the dose reaching cancer cells [49]. It was demonstrated that low-dose gemcitabine (GEM) enhances immunogenicity and natural killer (NK) cell-driven tumor immunity in lung cancer [112]. Low-dose GEM lipid nanocapsules showed ability to impact on myeloid derived suppressor cells (MDSCs) and potentiate cancer immunotherapy in lymphoma and melanoma-bearing mice [113]. In vitro experiments demonstrated that GEM-treated macrophages become tumoricidal, and postsurgical adjuvant GEM therapy in pancreatic ductal adenocarcinoma (PDAC) reprograms TAMs towards an M1-phenotype [98]. On the contrary, others have demonstrated that GEM promotes M2-polarization in pancreatic tumors [114,115].

Controversial observations, regarding macrophage polarization, have been also found in response to radiation therapy (RT). It has been suggested that M2 macrophages could be more resistant to X-ray radiation compared to M1, leading to an increase in the M2/M1 TAMs ratio in a preclinical model of glioblastoma [116]. In addition, irradiated TAMs could sustain cancer cell-invasion and angiogenesis [117]. However, others showed that low-dose RT leads to the release of DAMPs (e.g., dsRNA or tumor antigens) from the tumor cells inducing the reprogramming of macrophages towards an iNOS^+^/M1 phenotype [117,118,119]. In this case, the technological advances in the equipment used to apply the treatment result crucial to control the dose, time, and localization of the RT [120].

Within the last decades the controlled application of oncolytic viruses (OV) has emerged as an attractive therapeutic approach, owing to a preferential infection and killing of cancer cells, which results in innate antitumor immune responses and immunological memory [121]. In a similar manner to ICD or RT, OVs (i.e., influenza A, herpes simplex virus encoding GM-CSF, or adenovirus Delta24-RGD) induced the release of tumor-associated antigens and immunostimulatory signals, which promote TAMs polarization towards M1-like antitumor effectors [122]. Other approaches to induce ICD activation, such as RIG-1 activation, are currently being tested in preclinical and clinical studies alone or in combination with ICIs [123].

## 12. TLR or STING Signaling Activation to Reprogram TAMs

Toll-like receptors (TLRs) are innate immunity pattern recognition receptors expressed by antigen-presenting cells, including macrophages, which play a key role in orchestrating the immune response [3]. Nowadays, only imiquimod (R837; TLR7 agonist) is approved by the FDA (Food and Drug Adminstration) for cancer treatment. However, clinical trials have been performed for other TLR agonists, such as poly(I:C) (TLR3 agonist) [124,125]. We have recently demonstrated in vitro the superior efficacy of poly(I:C) versus R837 to stimulate M2-like or tumor-conditioned macrophages towards an M1-like antitumoral phenotype [126]. With the aim to improve the antitumoral efficacy of poly(I:C), we have also developed arginine-based poly(I:C)-loaded nanocomplexes to favor TAMs uptake and M1-antitumoral polarization. Macrophages exposed to poly(I:C)-nanocomplexes showed significant secretion of the T-cell attracting chemokines CXCL10 and CCL5 and improved ability to directly kill cancer cells [127]. Recently, the combination of CpG oligodeoxynucleotides (CpG; TLR9 agonist) with Ferumoxytol^®^ (FMT; an FDA approved drug for the treatment of iron deficiency) showed a synergistic antitumoral effect, mediated by an increased infiltration of M1-like macrophages (expressing F4/80 and iNOS) in a murine model of NSCLC [128]. A new hydrogel loaded with CpG and Doxorubicin showed also antitumoral efficacy, mediated by a decrease in M2-like TAMs and MDSCs, upon intratumoral implantation in a murine melanoma model [129]. Regarding the activation of extracellular TLRs, Diprovocim, a small drug agonist of TLR2, identified through a screening of molecules on macrophages, showed efficacy in combination with anti-PD-L1 antibodies in a melanoma model [130]. A similar antitumoral activity, mediated by TLR2 activation, was observed for the intratumoral injection of a modified glucomannan polysaccharide in murine models of sarcoma and melanoma [131].

Another innate immune pathway, involving stimulator of interferon genes (STING), a cytoplasmic DNA sensor anchored in the endoplasmic reticulum, has been used to reprogram TAMs through activation of IRF3 and type I interferon (IFN) genes [132,133]. STING agonists, such as cyclic dinucleotides (CDNs; e.g., cGAMP) or DMXAA, have been evaluated in preclinical and clinical trials [134,135]. In order to improve the delivery of CDNs to its intracellular target, Cheng et al. developed a liposomal formulation of cGAMP. In a murine model of breast cancer, cGAMP-liposomes induced TAMs reprograming, increased CD8+ T cell infiltration, reduced tumor growth, and prevented the formation of secondary tumors [136]. The inhalation of cGAMP-liposomes coated with phosphatidylserine also triggered type I IFN production in murine models of lung metastasis and showed long-term survival when combined with RT [137]. STING and TLR agonists have also been able to overcome resistance to monoclonal antibody therapies, through reprogramming of macrophages, by increasing the FcgR A:I (activatory: inhibitory) ratio, inducing proinflammatory cytokine responses and enhancing the mAb-mediated phagocytic activity [136]. In vivo, the STING agonists changed the FcγR A:I ratio and enhanced the efficacy of anti-CD20 mAb therapy [138]. Of note, the use of TLR or STING agonists, as adjuvants, alone, or in combination has been also evaluated in the context of cancer vaccination in preclinical and clinical trials [122,139].

## 13. Monoclonal Antibodies to Reprogram TAMs

As TAM targeting has been recognized important for the treatment of cancer, several monoclonal antibodies (mAb) have been investigated to manipulate macrophage recruitment or polarization into the tumor. Anti-CD40 antibodies have shown agonistic activity to reprogram TAMs resulting in effective antitumoral activity [140,141]. Macrophages and dendritic cells express on their surface CD40, a receptor of the TNF receptor family, which upon interaction with its ligand CD40L, mainly expressed by T cells, basophils, and mast cells, upregulates the expression of MHC (Major Histocompatibility Complex) molecules and the secretion of pro-inflammatory cytokines, thus promoting T cell activation [142]. The combination of anti-CD40 with anti-CSF-1R, which impairs the recruitment of new TAMs towards the tumor, was effective to treat “cold” preclinical tumor models, not responsive to immune checkpoint inhibitors (ICIs). The combination of these antibodies was able to turn “cold” into “hot” tumors, now responding to ICIs, through a decrease in immunosuppressive cells, TAMs reprogramming, activation of cytotoxic T cells and thus unleashing of potent antitumor immunity [143,144]. In clinical trials, anti-CD40 mAbs are being evaluated in combination with ICIs, chemotherapy or other targeted therapies [49]. A similar mechanism of action, promoting the anti-tumoral functions of macrophages in tumors, has been observed for anti-MARCO mAbs, in murine models of breast, colon cancer, and melanoma [145]. These antibodies target the pattern-recognition scavenger receptor, MARCO, which is overexpressed in TAMs and linked to poor prognosis in breast, lung, and hepatic cancer [146,147,148]. Bispecific antibodies, targeting angiopoietin-2 (Ang-2) and vascular endothelial growth factor (VEGF), showed TAMs reprogramming and delayed tumor growth in glioma murine models [149]. The combination of Ang-2/VEGF bispecific antibodies with 5-FU and irinotecan in colorectal cancer or with temozolomide in glioma, showed significant benefits versus the combination of anti-VEGF with chemotherapy [150,151].

An important approach, using mAbs to reprogram TAMs into antitumor effectors, consists of the manipulation of the CD47–SIRPα axis. The signal regulatory protein-α (SIRPα, also known as SHPS1), expressed on the surface of phagocytic cells, such as macrophages, interacts with CD47, expressed by the target cells, resulting in the inhibition of phagocytosis and thus acting as a “don’t eat me” signal for tissue homeostasis [152]. In preclinical cancer models, the pharmacological inhibition of CD47, overexpressed by cancer cells, restores the ability of macrophages to phagocyte and kill tumor cells [153,154,155]. Antibodies able to inhibit SIRPα showed satisfactory antitumoral activity in lung cancer models, however their effect was limited in time [156]. The sustained reprogramming of TAMs towards M1-antitumor effectors was achieved by the self-assembled combination of SIRPα-blocking antibodies with CSF-1R inhibitors. This combined therapy activates antitumor macrophages, by hindering the CD47-SIRPα ligation, while impairing recruitment of new TAMs by inhibition of CSF-1R [157,158]. Clinical trials using anti-CD47 mAbs or CD47-Fc fusion proteins are on-going for the treatment of hematological cancers or refractory solid tumors in combination with anti-PD-1 therapy or with anti-CD20 (Rituximab^®^) to target B cells [159].

## 14. Genetic and Epigenetic Intervention to Reprogram TAMs

M2 and M1 macrophages are characterized by distinct genetic programs. Thus, the therapeutic reprogramming of TAM genetic features towards antitumoral macrophages has been investigated using different methodological approaches: including the delivery of nucleic acids (i.e., RNAs), direct gene editing (i.e., CRISPR/Cas9 system), or even through manipulation of gene’s activity and expression at epigenetic level.

Interference RNAs, such as small interfering RNA (siRNA) or microRNAs (miRNA), can be used to silence the expression of immunosuppressive genes, while the administration of messenger RNA (mRNA) may be applied to activate the stimulatory pathways in macrophages to fight against the tumor. It is worth noting that delivery of RNAs as free molecules into cells comes with several issues, for instance concerning their biochemical properties (as large polyanions cannot cross easily the plasmatic membrane) and their biological features (nucleases can easily disrupt them). Thus, the implementation of NP-based delivery vehicles is investigated to hide and protect the RNA molecules until their entry in the cytosol. For example, charge-altering releasable transporters (CARTs) are positively charged NPs used to incorporate anionic mRNA molecules and to release them in the cytoplasm in response to the cellular pH [160]. CARTs were employed to deliver mRNA for CD70, OX40L, CD80, and CD86 (co-stimulatory proteins for T-cells), as well as IL-12 and IFN-γ (cytokines that enhance Th1 tumoricidal response) to macrophages in murine subcutaneous models of two tumors, where only one tumor was treated. Upon intra-peritumoral administration of CD80/86 + IL12 and OX40L mRNA-CARTs the mice showed a complete response at the treated tumor and systemic anti-tumor immune response, as seen by the regression of the untreated distal tumor [161]. Another approach was described using di-mannose-functionalized polymeric NPs to deliver towards TAMs two mRNAs encoding IRF5, an interferon regulatory factor, and IKKβ, a kinase that phosphorylates and activates IRF5. The intraperitoneal injection of these NPs in murine models of ovarian cancer was able to reprogram TAMs, and their antitumoral efficacy was also confirmed in murine models of glioma and lung metastasis [162].

Instead of mRNA, siRNAs were used to silence the expression of genes that control TAMs immunosuppression. Song et al. developed mannosylated dual pH-responsive NPs delivering two siRNAs against vascular endothelial growth factor (VEGF) and placental growth factor (PIGF), directed towards TAMs in murine models of breast cancer. The intravenous administration of these siRNA-NPs inhibited tumor-induced neoangiogenesis and lung metastasis [163]. Other dual-targeting NPs were designed, linking a scavenger receptor B type 1 (SR-B1) targeting peptide and an M2 macrophage binding peptide (M2-pep), to deliver anti-CSF-1R siRNA towards TAMs in murine models of subcutaneous melanoma [164]. These NPs led to a decrease in macrophage number in tumors, reduced tumor size, IL-10, and TGF-β, while an increase in CD8^+^ T cells [165]. A similar result was observed for siCCR2-NPs intravenously injected in orthotopic models of breast cancer [166]. These NPs may act on the monocyte precursors of TAMs, as the CCL2/CCR2 axis is the main responsible of monocyte recruitment towards tumors [3].

Gene silencing was also investigated using micro RNAs (miRNAs). miR-125a is upregulated in M1-macrophages and suppressed in M2 [167,168]. To revert this situation, Zhao et al. transduced in vitro bone-marrow derived macrophages (BMDMs) with a lentivirus overexpressing miR-125a and mixed them with Lewis lung cancer (LLC) cells prior to their subcutaneous injection in syngeneic mice. This approach allowed to correlate the antitumoral effects of injected miR-125a-BMDMs with M1-like polarization, characterized by higher iNOS and lower CD206 expression, resulting in antitumoral effects [169]. Later, Parayath et al. developed NPs to encapsulate and deliver miR-125b in KRAS/p53 murine models of NSCLC. This miRNA was responsible for increase in macrophage numbers in tumors and for rise in M1/M2 ratio [170]. Another approach consisted in the encapsulation of miR155 in lipid-coated calcium phosphonate NPs, conjugated with mannose to target CD206 on TAMs and with a pH-responsive coating to uncover the content of NPs once reaching the tumor acidic microenvironment. These miR155-NPs showed antitumoral effect in murine models of fibrosarcoma [171]. Finally, it is important to pay attention to the recently identified protumoral role of some miRNAs [172,173], which may require the development of strategies for their inhibition. To our best knowledge, no clinical trials have been initiated using RNA-based NPs towards TAMs.

Gene editing, consisting in DNA intervention, still presents difficulties with regard to both technological and ethical aspects. However, its possibilities for application in the clinic have been significantly accelerated since the discovery of the CRISPR/Cas9 system for precise gene editing of mammalian cells. The CRISPR/Cas9 approach was used ex vivo to prevent the expression of siglec-10 in primary monocytes [174] or the SIRP-α gene in murine macrophages (RAW 264.7) [175], with the aim to dismantle the siglec-10/CD24 or the SIRP-α/CD47 immune checkpoints, respectively, and improve the phagocytic activity of macrophages. Although the results are interesting, their application in preclinical cancer models remains to be tested. Gene editing, using novel lentiviral-based systems, was also investigated to transduce into primary macrophages both soluble transforming growth factor beta receptor II (sTβRII), to inhibit TGF-β and reduce immune suppression, and interleukin-21 (IL-21), to induce M1-like activation. These gene-edited macrophages were treated with GM-CSF and intratumorally injected in orthotopic glioma murine models, showing a continuous M1-antitumoral genetic programming for several weeks and ability to prevent tumor growth [176]. Despite their difficult application in the clinic, we foresee more investigations on the ex vivo preparation of M1-antitumoral gene-edited macrophages providing important information on their long-term efficacy and safety aspects.

As an alternative, the epigenetic manipulation of macrophages offers the possibility to manipulate the transcriptional machinery of the cells without direct intervention of their DNA or RNA. Histone deacetylases (HDACs) are a class of enzymes that remove acetyl groups from histones (proteins associated with chromatin), reducing the expression of affected genes. The intraperitoneal administration of TMP195, an inhibitor of HDAC7, in murine models of breast cancer (MMTV-PyMT) resulted in a major infiltration of CD11b+ myeloid cells and Mac-2 + mature macrophages inside the tumors showing upregulation of M1-genes. Furthermore, TMP195 treatment, not showing a direct effect on the cancer cells, resulted in immune-mediated killing of cancer cells and improved the efficacy of chemotherapy and anti-PD-1 in MMTV-PyMT tumor bearing mice [177]. Other epigenetic approaches to reprogram TAMs have relayed on intervention of histone methyl transferases or histone demethylases [178]. In addition, several ongoing clinical trials are studying the combination of epigenetic treatments with ICIs [179].

## 15. Metabolic Manipulation to Reprogram TAMs

The crosstalk between cancer cells and immune cells in the tumor microenvironment implies also changes in the metabolism of macrophages. TAMs present distinct glucose, lipid, amino acid, oxygen, and iron consumption, which support their protumoral and immunosuppressive properties to favor tumor growth. In experimental settings, it has been demonstrated that therapeutic intervention of metabolic pathways inside macrophages and/or in the tumor extracellular space can be applied to reprogram TAMs towards M1-like antitumoral macrophages [180,181].

With regard to energy metabolism, cancer cells with high glycolytic activity decrease the availability of glucose and induce an acidic microenvironment, which push TAMs towards an M2-like metabolism, strongly committed to oxidative phosphorylation and fatty acid oxidation, in contrast to M1-like macrophages, which present higher glucose uptake and aerobic glycolytic catabolism [182]. To alleviate hypoxia, hyaluronic acid NPs targeted towards TAMs have been designed to increase O_2_ production in the TME, showing inhibition of tumor growth and metastasis in 4T1 tumors [183,184]. Inactivation of HIF-1α by restoring miR-30c expression in macrophages showed antitumoral efficacy in gastric cancer [185]. In other reports, tumor oxygenation was improved by normalizing the tumor vasculature, through inhibition of VEGF or REDD1 in TAMs [186]. Blockade of glycolysis using 2-deoxy-D-glucose (2DG) was also used to abrogate TAMs ability to induce angiogenesis, extravasation, and EMT [187]. Another strategy to disrupt the M2-polarization pathways activated by lactate in TAMs consisted in the use of MEK/STAT3 inhibitors (i.e., selumetinib or static) [188].

Regarding the metabolism of lipids, fatty acid uptake and fatty acid oxidation (FAO) are downregulated in M1 macrophages, while FAO and mitochondrial activity are enhanced in M2 [189]. Linoleic acid (unsaturated fatty acid) rather than stearic acid (saturated fatty acid) promoted cytotoxic functions of macrophages towards cancer cells [181]. Etomoxir, an FAO inhibitor, blocked IL-4-induced M2 macrophage polarization [189], and EI-05, activator of the intracellular lipid chaperone E-FABP (epithelial fatty acid binding protein), led to the increase in IFN-β production by macrophages and improved antitumoral responses in murine models of breast cancer [190].

With regard to amino acids, altered L-arginine metabolism was one of the first markers identified to classify myeloid polarization [191]. Myeloid cells use distinct metabolic pathways to catabolize the essential L-arginine. M1-macrophages convert L-arginine to NO and L-citruline by inducible nitric oxide synthase (iNOS), while M2-macrophages skew arginine catabolism towards the production of ornithine and polyamines [191]. Upregulation of arginase 1 (Arg1) is pro-tumoral, while induction of iNOS presents a dual role, likely depending on amount of NO, type of tumor, and concomitant presence of ROS [192]. Blocking L-arginine uptake by tumor MDSCs impaired their protumoral properties in murine models of prostate cancer [193]. The effects of other amino acids on macrophage’s polarization was also studied. Extracellular adenosine, via adenosine receptors, supports protumoral functions of TAMs by altering their phagocytic activity, cytokine, and VEGF production [194]. The knockout of the adenosine receptor A2A in myeloid cells resulted in prevention of tumor growth and metastasis in melanoma tumor models [195]. Pharmacological or genetic deletion of glutamine synthase in macrophages reduced intracellular glutamine and increased succinate, showing M1-polarization properties and less metastasis in LLC models [196]. TAMs and MDSCs up-regulate indoleamine 2,3 dioxygenase (IDO), which converts tryptophan in kynurenines, favoring regulatory T-reg cells expansion [197], consequently several IDO inhibitors are being tested in clinical trials.

Finally, it is important to understand the role of iron metabolism in the polarization of macrophages, as iron homeostasis is required for DNA-synthesis, hematopoiesis, mitochondrial biogenesis, energy metabolism, and oxygen transport. M1-macrophages present a higher uptake, while M2-macrophages present a higher release of iron by ferroportin, enhanced heme catabolism by heme-oxygenase-1 (HO-1), and restricted iron retention by ferritin [198,199]. It was demonstrated that Ferumoxytol^®^ (iron-based NPs) increase intracellular iron in TAMs, inducing their M1-polarization in vitro and in vivo and showing therapeutic effects in breast cancer and NSCLC liver metastasis [200]. Suppression of the iron-releasing enzyme HO-1 promoted the expansion of M1-macrophages and reduced tumor growth in breast cancer models [201]. On the contrary, HO-1 has been found to be pro-inflammatory in chronic metabolic inflammation related to obesity [202], and in the context of colon cancer, increased HO-1 production by intestinal macrophages helps to resolve inflammation and prevents carcinogenesis [203]. These studies provide some insights to manipulate iron levels or HO-1 levels to control inflammation to treat cancer, or also other diseases.

These observations show a strict interplay between metabolism of TAMs and their immune functions that should be taken into account to design new strategies to intervene on macrophages within tumors, however this crosstalk remains to be further investigated [204].

## 16. Conclusions

The tumor microenvironment (TME) drives the success of antitumoral therapies and the outcome of patients with cancer. The targeting of TAMs, as the most abundant immune population in the TME, has been an important object of oncological studies. These studies, in different types of tumor, have provided clues about the molecular mechanisms and relevant markers for the pro-/anti-tumoral functions of macrophages, which can be now targeted. Taking advantage of this knowledge, the scientific community has focused its efforts on the reprogramming of macrophages to revert immunosuppression and to unleash their anti-tumoral functions. Consequently, a wide range of pharmacological strategies have been investigated, as described in this review, and many of them are now in clinical trials. These approaches are being tested as monotherapies, but mainly in combination with traditional chemotherapies or with new immunotherapies, such as anti-checkpoint inhibitors or adoptive cell transfer (i.e., CART cells). Furthermore, in the near future, we expect that new drug delivery approaches will help to improve significantly the efficacy of TAM reprogramming for the treatment of cancer.

## Figures and Tables

**Table 1:** Prognostic significance of TAMs and relevant markers in different human tumor types.

Tumor Type	Markers of TAMs	Prognostic Impact	References
Glioma	CD68, CD163, CD204	Bad	[73]
	IBA, CD204	Bad	[74]
	CD163/CCL3 ratio	Bad (if high ratio)	[75]
	CD68, CD163/AIF ratio	Bad (if high ratio)	[76]
Mesothelioma	CD68	Bad	[69,70]
	CD163	Bad	[72]
Lung cancer (NSCLC)	CD68	Good (in tumor islets/bad in tumor stroma)	[61]
	CD68/iNOS (for M1); CD68/CD163 (for M2)	Bad (if M2 > M1)	[77]
Pancreatic cancer	CD68, CD204	Bad	[78]
Breast cancer	CD163	Bad	[79]
	CD68, CD163	Bad	[80,81]
	iNOS	Good (if together with high CD8+ cells)	[17]
Colorectal cancer	CD68	Good (at the invasive front)	[82,83]
	Wnt5a, CD68	Bad	[84]
Melanoma	CD68, CD163	Bad (CD163 at tumor stroma and CD68 at invasive front)	[85]
Bladder cancer	CD68	Bad	[86]

TAM: tumor-associated macrophages; NSCLC: non-small-cell lung cancer.
